# Worsening angle closure glaucoma and choroidal detachments subsequent to closure of a carotid cavernous fistula

**DOI:** 10.1186/1471-2415-12-28

**Published:** 2012-07-28

**Authors:** Sumeer Thinda, Mark R Melson, Rachel W Kuchtey

**Affiliations:** 1 Vanderbilt Eye Institute, Vanderbilt University Medical Center, Nashville, TN, 37232, USA

## Abstract

**Background:**

Carotid cavernous fistulas are abnormal communications between the cavernous sinus and the external or internal carotid arteries. Although rare, closure of carotid cavernous fistulas can lead to immediate ocular complications. To our knowledge, our case represents the first report of worsening angle closure glaucoma and choroidal detachments over an extended period of two months subsequent to closure of a carotid cavernous fistula.

**Case presentation:**

A 70-year-old female with a history of primary angle closure glaucoma presented with 4 mm of proptosis, resistance to retropulsion, tortuous corkscrew blood vessels and an orbital bruit of the right eye. Diagnostic cerebral angiogram showed a small indirect Barrow type D right carotid cavernous fistula. Transarterial embolization was planned but repeat cerebral angiography prior to the procedure demonstrated spontaneous partial closure of the carotid cavernous fistula and the procedure was aborted. One month later, our patient was noted to have worsening vision and choroidal detachments of the right eye. She declined further testing and was thus started on self-administered manual carotid jugular compressions. One month later, she developed progressive worsening of her choroidal detachments and angle closure. She eventually opted for surgical intervention but repeat cerebral angiography showed significant thrombosis of the carotid cavernous fistula and no intervention was warranted. Examination two months later showed complete resolution of the choroidal detachments and open angles of both eyes.

**Conclusions:**

Our patient demonstrated worsening angle closure glaucoma and choroidal detachments after spontaneous closure of her carotid cavernous fistula had been noted. Ocular complications, including acute angle closure, have been reported to occur immediately after closure of carotid cavernous fistulas, but not over months as in our patient. It is imperative that individuals who have undergone apparent closure of a carotid cavernous fistula be carefully monitored for worsening ocular complications.

## Background

Carotid cavernous fistulas (CCF) are abnormal communications between the cavernous sinus and the external or internal carotid arteries [1]. Although rare, cases of ocular complications, including choroidal detachments and angle closure glaucoma (ACG), have been reported to occur immediately after closure of CCFs [2-4]. To our knowledge, our case represents the first report of worsening ACG and choroidal detachments subsequent to CCF closure over an extended period of two months.

## Case presentation

A 70-year-old female with a history of primary angle closure glaucoma (PACG) status post laser peripheral iridotomy (PI) of both eyes (OU) presented for further management. She complained of discomfort and redness of the right eye (OD). Past medical history was significant for hypertension, gastroesophageal reflux disease and a cerebrovascular accident 15 years prior. Medications included atenolol, alprazolam, omeprazole, aspirin and topical prednisolone four times a day OD. The duration of topical prednisolone treatment was approximately 1 week prior to her presentation to our institution. The treatment was deemed necessary by the referring physician for her complaint of discomfort and redness. No other topical medications were given. Intraocular pressure (IOP) was 22 OD and 16 of the left eye (OS). Exam showed mild injection OD, shallow anterior chambers, patent PIs and cataracts OU. Gonioscopy demonstrated narrow angles with extensive peripheral anterior synechiae OU. Dilated fundus exam showed increased vessel tortuosity OU.

Given the significant narrow angles despite patent PIs OU, cataract extraction with intraocular lens implantation OU was performed. She was treated postoperatively with topical moxifloxacin, nepafenac and prednisolone for a month. She did not receive dorzolamide or other sulfa derivatives. Postoperatively, the anterior chambers deepened and the angles opened significantly. IOP was noted to be 19 OD and 18 OS. No hypotony was detected throughout the entire course.

Two months after cataract surgery, she developed 4 mm of proptosis, resistance to retropulsion, tortuous corkscrew blood vessels and an orbital bruit OD. Gonioscopy revealed the recurrence of narrow angles OD.

**Figure 1:**
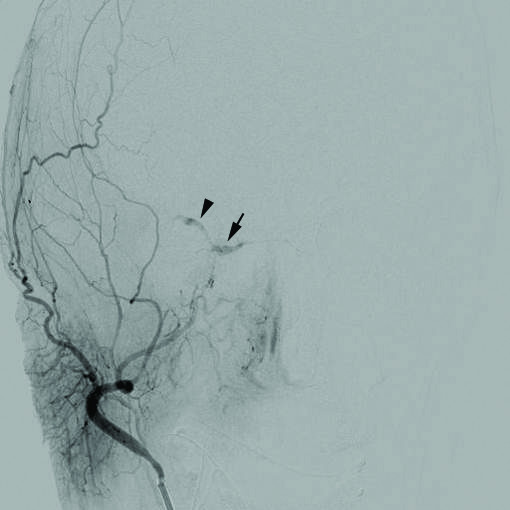
**Cerebral angiogram showing a small indirect Barrow type D right carotid cavernous fistula.** Selective injection of the external carotid artery demonstrates filling of the cavernous sinus (arrow) and retrograde drainage into the right superior ophthalmic vein (arrowhead).

A CCF was suspected and both computed tomography (CT) of the orbits with and without contrast and computed tomography angiography (CTA) of the head were performed. The CT orbits showed possible asymmetry of the superior ophthalmic veins (SOV). The CTA head showed atherosclerotic disease within the distal cavernous segments of the internal carotid arteries. As neither imaging modality was completely diagnostic and high suspicion for a CCF remained (based on the clinical examination findings), a six vessel cerebral angiogram was performed. The diagnostic cerebral angiogram showed a small indirect Barrow type D right carotid cavernous fistula with retrograde drainage into the right SOV (Figure 1). Early filling of the right SOV was seen on right internal carotid artery injection; however, there were no feeders large enough to be actually visualized. On injection of the right external carotid artery, there was filling of the cavernous sinus via small branches of the right accessory meningeal artery. Our patient did not have a suitable endovascular corridor to the CCF via the petrosal sinuses, therefore transfemoral venous embolization did not appear possible. The plan was for transarterial embolization and if satisfactory occlusion could not be achieved from embolization of the right accessory meningeal artery feeder, then an alternative approach through the right SOV was to be considered. When diagnostic cerebral angiography was repeated prior to the planed embolization procedure, it was noted that she had undergone spontaneous partial closure of her CCF and the procedure was aborted.

**Figure 2:**
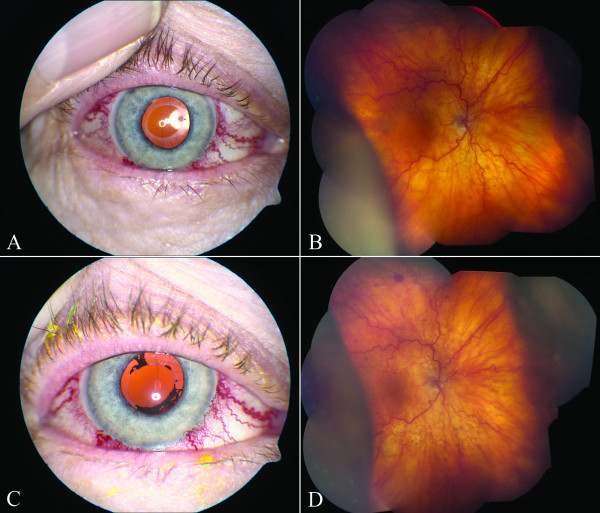
**Photographs of the right eye taken one month (A, B) and two months (C, D) after closure of the CCF.** (**A**) External photograph showing corkscrew blood vessels. (**B**) Fundus photograph showing intraretinal hemorrhages, tortuous retinal vessels and choroidal detachments. (**C**) External photograph showing corkscrew blood vessels and iris pigment on the anterior capsule. (**D**) Fundus photograph showing worsening choroidal detachments.

One month later, our patient developed worsening vision and was noted to have a choroidal detachment OD (Figures 2A and B). She declined further angiographic testing and was thus started on self-administered manual carotid jugular compressions. One month later, she was noted to have progressive worsening of her choroidal detachments and angle closure (Figure 2 C and D).

She eventually opted for repeat surgical intervention but when diagnostic cerebral angiography was performed prior to the embolization procedure, significant thrombosis of the CCF was noted. There was virtually no filling of the SOV on angiography and no intervention was warranted. Examination two months later showed complete resolution of her choroidal detachments and open angles OU.

## Conclusions

Our patient demonstrated worsening of her ACG and choroidal detachments after spontaneous closure of her CCF had been noted. Her signs and symptoms worsened over a two month period as her CCF first partially closed spontaneously and then completely closed with self-administered manual carotid jugular compressions.

Ocular complications, including acute angle closure, have been reported to occur immediately after closure of CCFs, but not over months as in our patient [2-4]. The involved mechanism is unclear but it has been postulated that perhaps thrombosis of the SOV in the absence of sufficient collateral drainage leads to worsening of signs and symptoms [2]. Once collaterals develop or expand, the ocular manifestations eventually improve [2].

Our patient also had a history of underlying PACG that may have predisposed her to the development of choroidal detachments. Studies have shown an association of PACG with underlying uveal effusion [5-7]. It has been proposed that individuals with PACG may have a dysfunctional ability to regulate choroidal thickness [6]. Patients with CCFs have abnormal venous drainage that results in subsequent orbital congestion [8]. The underlying propensity of patients with PACG to accumulate fluid in the choroidal space may be exacerbated by a CCF and result in the development of a choroidal detachment. Choroidal detachments are relatively rare in the setting of CCFs [8] and our patient was likely predisposed to this complication as a result of her underlying PACG and uveal effusion.

It is imperative that individuals who have undergone apparent closure of a CCF be carefully monitored for worsening ocular complications. In addition, PACG may be a risk factor for the development of choroidal detachments in the setting of a CCF.

## Consent

Written informed consent was obtained from the patient for publication of this case report and any accompanying images. A copy of the written consent is available for review by the Editor-in-Chief of this journal.

## Competing interests

The authors declare that they have no competing interests.

## Authors’ contributions

ST conducted a literature search and drafted the manuscript. RWK conceived the idea for the manuscript, conducted a literature search and critically revised the manuscript. MRM critically revised the manuscript. All authors read and approved the final manuscript.

## Pre-publication history

The pre-publication history for this paper can be accessed here:

http://www.biomedcentral.com/1471-2415/12/28/prepub
